# Astrocyte Calcium Responses to Sensory Input: Influence of Circuit Organization and Experimental Factors

**DOI:** 10.3389/fncir.2017.00016

**Published:** 2017-03-22

**Authors:** Mónica López-Hidalgo, Vered Kellner, James Schummers

**Affiliations:** Max Planck Florida Institute for NeuroscienceJupiter, FL, USA

**Keywords:** astrocyte, visual cortex, calcium, *in vivo* imaging

## Introduction

Astrocytes are important partners to neurons due to their involvement in homeostatic functions (Olsen et al., 2015; Verkhratsky et al., 2015), synaptic transmission (Perea and Araque, 2007; Di Castro et al., 2011; Panatier et al., 2011), synaptic plasticity (Perea and Araque, 2007; Min and Nevian, 2012; Valtcheva and Venance, 2016), and cognitive functions (Han et al., 2012; López-Hidalgo et al., 2012; Lee et al., 2014; Lima et al., 2014; Matos et al., 2015). These essential functions involve activation of calcium signaling pathways within the cytosol of the astrocyte, which can be activated intrinsically (Nett et al., 2002; Srinivasan et al., 2015; Rungta et al., 2016), or in response to neuronal activity (Di Castro et al., 2011; Haustein et al., 2014). Defining the quantitative relationship between neuronal activity and astrocyte calcium signaling has proven difficult. Early results in cultured astroglial cells demonstrated that neuronal activity can trigger calcium events which exhibit a number of distinct properties, including oscillations and traveling waves (Cornell-Bell et al., 1990; Parpura et al., 1994; Dani and Smith, 1995), which propagate throughout gap-junction coupled networks of cells, leading to the notion that astrocytes function as a syncytium (Finkbeiner, 1992; Giaume and Venance, 1998). Subsequent studies of astrocytes *in situ*, primarily in acute hippocampal slices, painted a different picture of astrocyte calcium activity; many small events were localized to individual branches, global cell-wide events were rare, and intercellular waves were very sporadic (Nett et al., 2002; Fiacco and McCarthy, 2004; Shigetomi et al., 2013a; Haustein et al., 2014). It was soon recognized that variations in tissue preparation, stimulation protocol, and other experimental factors might play a role in these differences. An important realization was that astrocytes readily adapt to surrounding conditions, and that even subtle experimental changes in physiological conditions can have profound effects on astrocyte calcium signaling (Takano et al., 2014; Mola et al., 2016).

It was hoped that some of these issues might be resolved by measuring astrocyte calcium activity *in vivo* during physiological activation of neuronal circuits, which was made possible with advances in both indicator labeling and imaging technologies (Denk et al., 1990; Stosiek et al., 2003; Nimmerjahn et al., 2004; Helmchen and Denk, 2005; Shigetomi et al., 2013b). Most *in vivo* studies of astrocytes have attempted to activate astrocyte calcium pathways by driving neuronal activity with sensory stimulation. Sensory-evoked calcium responses in astrocytes have been shown in the spinal cord (Sekiguchi et al., 2016), olfactory bulb (Petzold et al., 2008; Otsu et al., 2015), somatosensory cortex (Winship et al., 2007; Schulz et al., 2012; Ghosh et al., 2013; Zhang et al., 2016), barrel cortex (Wang et al., 2006), and visual cortex (Schummers et al., 2008).

However, studies with seemingly similar experimental design have led to different conclusions. While early studies reported robust astrocyte calcium activity in response to sensory stimulation (Wang et al., 2006; Schummers et al., 2008), recent studies have shown weak, sporadic, or non-existent responses to sensory stimulation in both visual (Bonder and McCarthy, 2014; Paukert et al., 2014; Asada et al., 2015) and somatosensory (Ding et al., 2013; Nizar et al., 2013) cortex. Interestingly, several of these studies noted stronger responses to neuromodulators than to sensory stimulation (Chen et al., 2012; Ding et al., 2013; Paukert et al., 2014). Taken together with the recent demonstration that mGluRs—initially thought to be responsible for neuronal-driven responses—may not be expressed in adult astrocytes (Sun et al., 2013), confusion has emerged as to whether astrocytes respond robustly to local synaptic activity *in vivo*.

Here, we attempt to reconcile the seemingly contradictory results in the literature and to synthesize an understanding of astrocyte calcium signaling that incorporates observations in different experimental preparations. We summarize fundamental differences in the neuronal circuit and astrocyte organization, sensory-evoked neuronal dynamics as well as technical issues involved in these studies that play important roles in the different patterns of astrocyte responses. We argue that apparently different results can be reconciled by considering these factors. Altogether, we propose a synthesis of the existing literature that astrocytes integrate signals from a variety of sources, including local synaptic activity and neuromodulators, but only generate calcium responses subject to a relatively high threshold. It remains unknown whether this threshold applies equally to somatic and subcellular responses in more distal portions of astrocyte processes, though *in vitro* responses to electrical stimulation suggest a similar threshold in both compartments (Haustein et al., 2014). Since most *in vivo* studies have focused on somatic or global responses, we will focus mostly on these, but we note that subcellular responses are a topic of ongoing investigation and may provide additional insight into neuron-astrocyte communication.

## Visual system in ferrets and rodents: neuronal circuits

The most disparate results have come in studies of visual cortical astrocytes. One difference between the contradictory studies is the use of different species. While visually-evoked calcium responses in ferret visual cortex astrocytes are robust and highly tuned to visual stimuli (Schummers et al., 2008), in mice, visual responses in astrocytes are generally reported to be weak, unreliable, or sparse (Bonder and McCarthy, 2014; Paukert et al., 2014; Asada et al., 2015; though see Chen et al., 2012). An understanding of the differences between the functional organization of rodent and ferret visual cortex may shed light on this apparent conflict.

**Figure 1:**
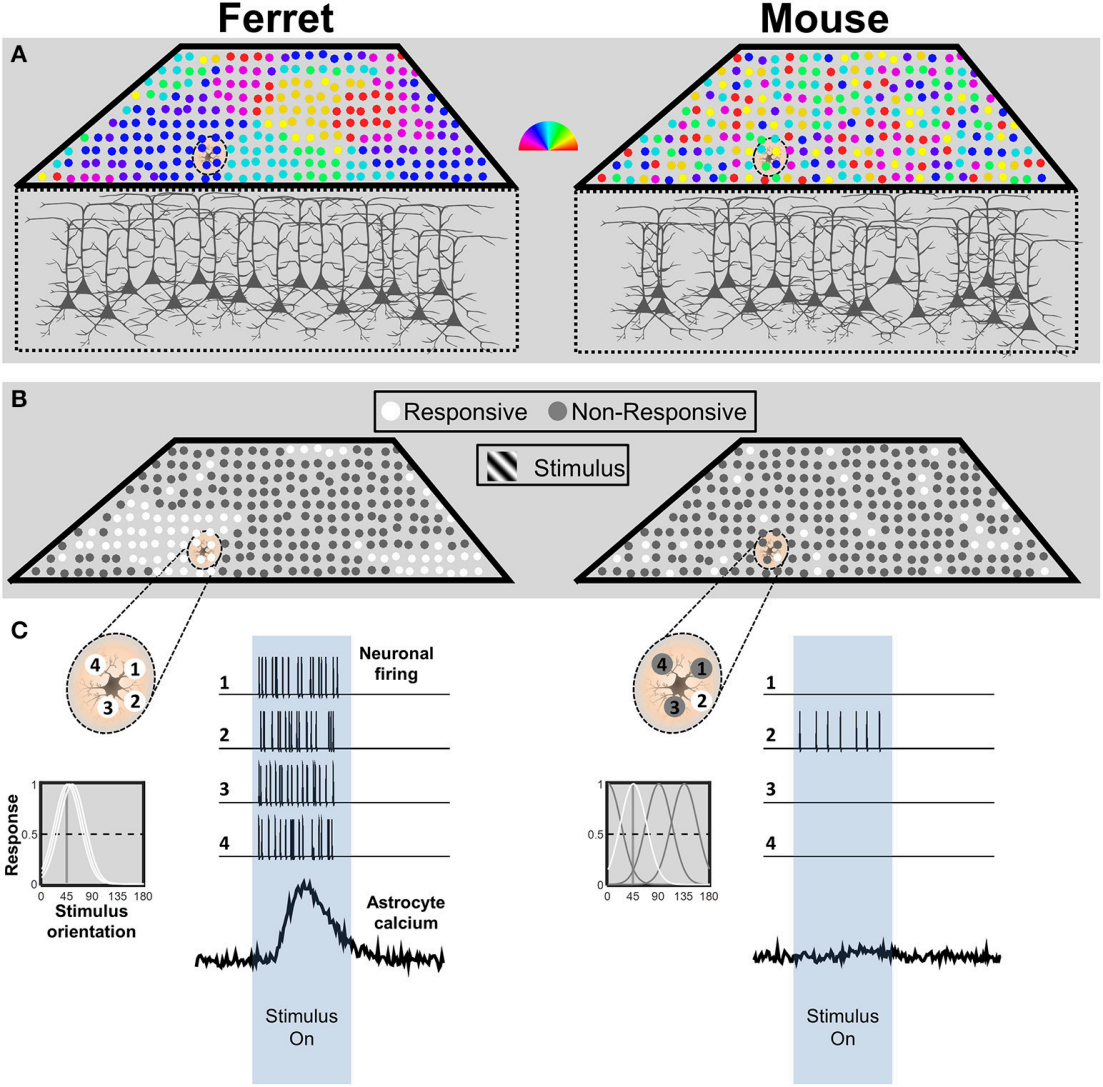
**Schematic representation of neuronal response patterns in mouse and ferret visual cortex. (A)** The orientation preference map for a cube of cortex in both species. Each neuron is represented by a circle, and its preferred orientation in indicated by the pseudocolor scalebar. Due to the salt-and-pepper organization in the mouse, the astrocyte is in contact with neurons with multiple preferred orientations. In contrast, the astrocyte in ferret visual cortex is surrounded by neurons with similar preferred orientations. **(B)** Response patterns to stimulation with an oriented grating stimulus. The model assumes equivalent or no neuromodulatory input. The example astrocyte in mouse cortex only contacts a single active neuron (white circle), whereas the astrocyte in ferret cortex contacts four active neurons. **(C)** Schematic depiction of an astrocyte from ferret (left) and mouse (right) visual cortex and four neurons located within its territory (top left side of each panel). The corresponding tuning curves for each neuronal response are depicted below. Tuning curves of neurons that would produce a strong (>50%) response to a 45° stimulus are drawn in white. Simulated spike trains corresponding to the four neurons and calcium response patterns of the astrocyte are depicted in response to the stimulus (right side of each panel). n.b. this simplified representation is not intended to imply that astrocytes respond directly to the activity of neuronal somata. The ratios of synaptic activity between the mouse and ferret would be expected to be similar.

Given that astrocyte calcium responses are driven by synaptic activity, it is important to consider the spatio-temporal patterns of neuronal activity that a visual stimulus, such as a grating, would be expected to evoke in each species. In primates and carnivores including ferrets, primary visual cortex (V1) is organized in vertical columns according to preferred orientation (Figure 1A, left; Hubel and Wiesel, 1962; Grinvald et al., 1986; Chapman et al., 1996). Rodents do not have this organization; preferred orientation is spatially random, in a so-called salt-and-pepper arrangement (Figure 1A, right; Dräger, 1975; Mangini and Pearlman, 1980; Ohki et al., 2005; Ohki and Reid, 2007; Kondo et al., 2016; Ringach et al., 2016).

Owing to the columnar organization of orientation preference in ferret, we would expect the majority of neurons within the territory of an astrocyte to respond together to a grating of the preferred orientation (Figure 1B, left). In contrast, based on the typical half-width of mouse tuning curves (~25°; Niell and Stryker, 2008), a simulation of the responses in a salt-and-pepper cortex to a particular orientation (Figure 1B, right), would drive responses in only a small fraction of the neurons in the territory of a mouse astrocyte (~27% of neurons would have a strong response, defined as >50% of max response; Figure 1C, right). Furthermore, firing rates evoked by a grating stimulus are nearly three times higher in ferret (~19 Hz; M Popović and SD Van Hooser, personal communication) than in mouse visual cortex (~7 Hz; Niell and Stryker, 2008; Durand et al., 2016). Thus, during the presentation of an oriented grating, an astrocyte in ferret visual cortex would receive input from more neurons (four-fold) which fire more action potentials (three-fold), which means 12 times more synaptic activity than an astrocyte in mouse visual cortex (See Figure 1C). Considering that astrocyte responses are thought to derive from transmitter spillover (Patrushev et al., 2013), it is likely that this would result in a considerable difference in the amount of transmitter available to astrocyte receptors or transporters. The combined difference in synapse number and firing rate is likely to have a dramatic effect on determining whether the integrated activity reaches threshold to activate astrocyte calcium signaling.

One might then ask: if astrocyte calcium responses depend on such strong concerted activation, would they ever be activated in mouse visual cortex? In other words, are the types of responses observed in ferret cortex the exception, rather than the rule? We would argue that these differences are a consequence of the experimental approach to studying visual cortex, which does not take into account important differences in the functional organization of the visual systems between the species. In particular, several lines of evidence suggest that V1 might not serve the same function in the visual hierarchy in rodents as in carnivores. In cats, ferrets and primates, visual information from the eye is processed in the retinal ganglion cells and most of the projections are sent to the lateral geniculate nucleus (LGN) which in turn drives neurons in V1 (Reid and Alonso, 1995; Hirsch et al., 1998; Alonso et al., 2001). In rodents, only 50% of retinal ganglion cells reach the visual cortex through the LGN (Martin, 1986; Gauvain and Murphy, 2015; Ellis et al., 2016). Instead, the majority of these projections (~70–90%) reach the superior colliculus (Linden and Perry, 1983a,b; Hofbauer and Dräger, 1985; Ellis et al., 2016), where in fact, orientation maps have been observed (Ahmadlou and Heimel, 2015; Feinberg and Meister, 2015; Inayat et al., 2015). This suggests the possibility that substantial processing in rodents occurs in subcortical structures and that V1 could serve as a higher-order visual area. Consistent with this, the activity of V1 neurons in rodents reflects multimodal processing of visual and locomotor signals (Niell and Stryker, 2010; Keller et al., 2012; Ayaz et al., 2013; Saleem et al., 2013). Locomotion increases activity of neurons in V1 and also corresponds to different brain states, including different electroencephalogram (EEG) states (Ayaz et al., 2013; Saleem et al., 2013) and neuromodulator tone (Niell and Stryker, 2010). Either of these could affect astrocyte responsiveness. If we consider astrocytes as integrators of neuronal activity, then these results could explain why mouse V1 astrocytes are not responsive to visual stimulation alone in stationary resting or sedated conditions.

If indeed species differences in neuronal circuit organization account for observed species differences in visual cortical astrocyte responses, can we learn anything from other sensory systems, in which rodents have a spatially organized stimulus representation? In the olfactory bulb, rodents have a clear organization over the different layers of the bulb and odors are mapped onto different glomeruli (Stewart et al., 1979; Greer et al., 1982; Lancet et al., 1982). Thus, presentation of an odor elicits concerted responses from local populations of neurons (Uchida et al., 2000, 2014) and calcium elevations in astrocytes also *in vivo* (Petzold et al., 2008; Otsu et al., 2015). In the somatosensory cortex, there is a precise topographic representation of the body surface, including the whisker field, which is often referred to as barrel cortex (Woolsey and Van der Loos, 1970). In the rodent somatosensory cortex, responses of astrocytes to sensory stimulation have been readily observed *in vivo* (Wang et al., 2006; Takata et al., 2011; Lind et al., 2013; Stobart et al., 2016) but see (Ding et al., 2013; Nizar et al., 2013). These comparisons lend support to the notion that the difference between astrocyte responses to visual stimuli in ferrets and mice is not necessarily species dependent but rather is determined by the organization of the neuronal circuitry in which it is embedded.

## Visual system in ferrets and rodents: astrocyte organization

It remains possible that some observed differences are also a reflection of astrocyte specializations. Astrocyte diversity across brain regions and species is well-documented, and it is commonly thought that astrocyte spatial distribution and morphology are well-suited to accomplish their circuit-specific functions (De Saint Jan and Westbrook, 2005; Houades et al., 2008; Roux et al., 2011). Rodent cortical astrocytes are small cells with processes that tile gray matter of the brain (Bushong et al., 2002; Ogata and Kosaka, 2002). Each astrocyte covers ~75,000 μm^3^ of the gray matter in the hippocampus (Ogata and Kosaka, 2002; Bushong et al., 2002) and 66,000 μm^3^ in visual cortex (López-Hidalgo et al., 2016). Here, astrocytes establish exclusive territories so their processes overlap with their neighbors by <5% (López-Hidalgo et al., 2016).

Astrocytes from ferret visual cortex are considerably different from their counterparts in rodents. In ferrets, astrocytes are twice as big (~120,000 vs. 66,000 μm^3^), less spherical and have a wide variety of shapes (López-Hidalgo et al., 2016). Their processes extend up to 30 μm away from the soma and they overlap with the processes of 6–8 neighboring astrocytes and each astrocyte shares almost 50% of its territory with the processes of its neighbors (López-Hidalgo et al., 2016). Although, the functional consequence of exclusive territories is unclear, a larger astrocyte would interact with a larger number of synchronously activated neurons. Assuming astrocytes are integrators of neuronal activity with a threshold amount of activity needed to elicit calcium responses, then more neuronal activity within its territory would facilitate reaching threshold.

## Technical considerations

Despite the arguments laid out above, there remain inconsistencies in the literature that are difficult to attribute solely to these factors. It is worth considering what other factors might give rise to different experimental outcomes. Astrocytes play an important role in physiological homeostasis of the nervous system and are extremely sensitive to a host of environmental factors including physical insult, pH, temperature, extracellular ionic composition, and many others (Schipke et al., 2008; Verkhratsky et al., 2015; Mola et al., 2016). As a result, numerous subtle experimental factors can have important effects on the functional state of astrocytes, and lead to dramatically different results under seemingly similar experimental conditions.

Nearly all *in vivo* calcium imaging experiments of astrocytes involve making a craniotomy (and sometimes a durotomy), which necessarily risks insult or alteration of the physiological conditions at the brain surface. In the mouse, the preparation of the cranial window is particularly delicate. Due to the small distance between the skull and the brain, heating during drilling of the skull and brain compression during indentation of the thinned skull are a common source of variability from preparation to preparation. The material that contacts the brain surface can also have unintended impact on the extracellular milieu, with unanticipated consequences. In our experience, filling the craniotomy with agar dissolved in normal saline results in little to no astrocyte activity, whereas ACSF with proper ionic composition, pH and osmolarity leads to better results. An inert silicone plug, which is impenetrable by CSF, leads to more reliable astrocyte activity still (JS unpublished observations).

Another important factor is that under typical conditions, the brain temperature under a craniotomy in a mouse falls to ~29°C (Schipke et al., 2008; Takata and Hirase, 2008; Kalmbach and Waters, 2012). This affects many biological processes to which astrocytes are likely to be sensitive. In particular, the frequency band of local potentials and the UP and DOWN state transitions were shown to depend on cortical temperature (Kalmbach and Waters, 2012). The authors' experience is that without actively maintaining the brain temperature near physiological temperature (Runyan et al., 2010), visually-evoked responses are often difficult to detect in neurons as well.

It is difficult to assess or predict the effects of these various factors on astrocyte physiology or calcium activity. Due to their sensitivity to so many factors, it is important to recognize that astrocyte activity is likely to be influenced to a much larger extent than neuronal activity. Because of the apparent high threshold for activating calcium signaling pathways, even small changes in neuronal activity levels may have a large impact on astrocyte activity.

## Conclusion

Here, we have reviewed differences in the neuronal circuit and astrocyte organization in rodents vs. ferret visual cortex as well as methodological aspects that can explain the controversies in the literature regarding visual-evoked responses in astrocytes. We argue that one fundamental difference derives from the columnar vs. salt-and-pepper organization. In particular, visual stimulation with an oriented grating will elicit small, short-duration responses from a small proportion of synapses within the territory of an astrocyte in rodents, whereas the same stimulus will elicit robust, prolonged responses from a large fraction of the synapses within the territory of an astrocyte in ferret visual cortex.

We have emphasized these species differences in the visual cortex in part to clarify some discrepancies in the literature, but also as a starting point for understanding the general principles that govern the transfer of neuronal activity patterns to astrocyte calcium signaling events. Numerous lines of evidence suggest that the total amount of integrated local synaptic activity is necessary to exceed the threshold enabling calcium responses. This possibility is supported by the observation that astrocyte responses have been more reliably observed in rodents in areas with a spatially clustered sensory map, such as barrel cortex and olfactory bulb. A number of factors likely combine to set this threshold. Some of these may be considered physiologically relevant, such as neuromodulatory tone (Paukert et al., 2014), which can lower the threshold for synaptic activation either globally during alertness or arousal cues, or perhaps locally during learning or attentional states related to specific cortical areas. Other factors should likely be considered artifactual, owing to surgical or anesthesia conditions that put the cortex too far from the normal physiological state for the usual activation pathways to be activated.

Another important consideration is the spatial scale and localization of astrocyte calcium signals. Some *in vitro* experiments have indicated that small scale responses in the branches of astrocyte are responsive to low levels of neural activity (Di Castro et al., 2011; Panatier et al., 2011), whereas others have shown a similar sensitivity in branches and somata (Haustein et al., 2014). Thus, it remains an open question whether astrocyte processes have a lower threshold for sensory-evoked responses. Different methods for imaging calcium in astrocytes offer different resolutions for detecting subcellular calcium events in astrocytes. Many *in vivo* studies have used labeling with organic dyes that only afford limited resolution for calcium events that are not somatic. Few studies have systematically studied subcellular calcium sensory responses *in vivo* (Asada et al., 2015; Stobart et al., 2016). It is possible that oriented stimuli would elicit highly localized responses in mouse cortical astrocytes, which might have been more difficult to detect (though see; Bonder and McCarthy, 2014; Asada et al., 2015).

In conclusion, we have highlighted here several aspects which could explain the differences between astrocyte calcium responses to visual stimuli measured in ferret and rodent visual cortex by us and others. We suggest that these differences reveal an important aspect of astrocyte signaling. Astrocytes require a minimum amount of neuronal activity to respond to sensory stimuli *in vivo*. This reinforces the idea that astrocytes are perhaps not involved in the millisecond level of perception, as neurons are, but rather have a role in plasticity or synchrony of neuronal activity, although this has yet to be shown *in vivo*.

## Author contributions

ML, VK, and JS contributed to the conception and writing of the paper.

## Funding

This work was supported by the National Eye Institute grant R01EY026977 (JS) and the Max Planck Florida Institute for Neuroscience.

### Conflict of interest statement

The authors declare that the research was conducted in the absence of any commercial or financial relationships that could be construed as a potential conflict of interest.
